# 5xFAD/*Abi3^S209F^* mice display distinct age‐dependent effects on amyloid beta pathology and microglia dynamics

**DOI:** 10.1002/alz.089879

**Published:** 2025-01-03

**Authors:** Claire Ann Butler, Katie O'Gara, Adrian Mendoza‐Arvilla, Giedre Milinkeviciute, Celia Da Cunha, Shimako Kawauchi, Narges Rezaie, Heidi Yahan Liang, Annie Thach, Shuling Wang, Sherilyn Collins, Amber Walker, Kai‐Xuan Shi, Jonathan Neumann, Angela Gomez‐Arboledas, Andrea J Tenner, Frank M LaFerla, Ali Mortazavi, Grant R MacGregor, Kim N. Green

**Affiliations:** ^1^ University of California, Irvine, Irvine, CA USA

## Abstract

**Background:**

In several large genome‐wide association studies (GWAS), genetic polymorphisms of *Abi3* have been identified as a risk factor for late‐onset Alzheimer’s Disease (LOAD). ABI3 along with ABI1 and ABI2, regulate the formation of the WAVE complex which in turn, regulates actin dynamics. ABI3 is highly expressed in microglia in the brain, however, the function of ABI3 in microglia is relatively unknown. In recent studies knock‐out of ABI3 has been shown to exacerbate amyloid beta (Aβ) pathology and associated inflammation. To date, there have been no studies on variant specific effects on amyloid beta pathology.

**Methods:**

To study the effects of ABI3 on the development of Alzheimer’s disease (AD) relevant pathologies we introduced an equivalent coding sequence change that has been identified as a risk variant for LOAD (S212F) into the C57BL/6 mouse genome using CRISPR/Cas9 (S209F). We set out to characterize the *Abi3^S209^* ^F^ variant and investigate its impact on AD pathology when crossed with 5xFAD transgenic mice, generating four distinct groups: WT, *Abi3^S209^* ^F^ homozygous, 5xFAD, and 5xFAD/*Abi3^S209^* ^F^ homozygous. Characterization was performed using histological staining and biochemical approaches at 4‐, 12‐ and 18‐months of age.

**Results:**

5xFAD/*Abi3^S209^* ^F^ mice displayed an age‐related decrease in dense core‐Aβ plaque burden, characterized by reduced Thioflavin‐S staining, accompanied by changes in fibrillar Aβ (OC) staining, in confocal images of both subiculum and visual cortical regions of 5xFAD/*Abi3^S209F^* mice compared to 5xFAD mice. Broadly, microglial numbers mimic plaque load, however by 18‐months of age 5xFAD/*Abi3^S209F^* mice display dramatic reductions in IBA1+ microglia number – to levels comparable to WT/*Abi3^S209F^* homozygous mice, suggesting a general loss of plaque associated microglia. By 12 months of age, *Abi3^S209F^* homozygous and 5xFAD/ *Abi3^S209F^* mice displayed increased synaptic density, independent of pathology in visual cortex, subiculum and CA1 regions.

**Conclusions:**

Together, these results characterize the effects of the *Abi3^S209F^* missense mutation on 5xFAD‐mediated pathology, specifically in Aβ plaque development, glial morphology, and synapses. Our data suggests that this mutation may affect microglia dynamics in an age‐dependent manner and may result in dysfunctional microglia at later stages of disease.

